# Medical Legislation

**Published:** 1886-07

**Authors:** M. M. Myers

**Affiliations:** Krohne, Lyons P. O., Texas


### CORRESPONDENCE.

### MEDICAL LEGISLATION.

KROHNE, LYONS P. O., Texas, July 10, 1886.

Editor Daniel's Texas Medical Journal:

THE Texas State Medical Association, which met at Dallas in April, took no action regarding medical legislation, on the grounds that the people were the party who needed protection, and therefore the people must ask for protection.

The people do not understand the matter; they do not appreciate their position in the premises. I have talked to numbers on this subject often, and have heard prominent citizens discuss this subject, even within the last month, and they take the ground that the doctors should look after the protection of the people against quackery; that they alone are qualified to know who are competent and who are not, and that it is their duty to see that some means be provided to put an end to this fraud. I have seen a woman die from post partum hemorrhage (adherent placenta with hour glass contraction) for the want of timely treatment. We had one of those "doctors" in this locality, who had a case of "double croup," and another bright star of this profession, who had a case of a woman who "vomited her liver" and "passed her spleen off through the bowels and died." These self-styled doctors are considered a part of the medical profession, and put upon an equality with regular physicians by the laity; and whenever these cases are made public, the profession then becomes an object of ridicule; and while some make the doctors a laughing stock, the more thoughtful take a more serious view of the matter, and blame them for not putting a stop to such glaring frauds.

Now, what are we to do? Shall we sit still and keep our mouths shut and be a party to fraud; and, in some instances, to murder? No, sir; we must move in this matter; and, as some one said at Dallas, we must make our power felt; and then, and not until then, will the medical profession receive that recognition and support from the law-making power that its responsible position and dignity demand, and have a right to command, at the hands of our representatives.

How shall we do this? I will suggest the first step in this direction. Let every physician use his influence with the members of the Legislature from his own section. Let Dr. Acheson's Pamphlet on Texas Quackery be put in the hands of every member of the Texas Legislature. Let them know what quackery is doing for Texas. Talk this matter up in the "primaries," and demand from candidates their support, and refuse to vote for any man who ignores the medical profession.

I was approached by a gentleman a few days ago and informed that he would soon announce himself for the Legislature. I informed him that my vote would be cast and my influence be used for no man, unless the rights of the medical profession were fully recognized. He promised his influence and support. Now, let every physician do this, and we will soon see that we can do something to secure the rights and raise the dignity of the profession in Texas. Make the Legislature acquainted with the frauds, malpractice, and even murders committed by these incompetents; and instead of ourselves being tacitly *particeps criminis*, let them take the blame upon themselves, in case they fail to act in obedience to justice and the demands of humanity. We do not ask that this or that school of medicine be forbidden to practice medicine in Texas; but we think it reasonable to demand that every man who wishes to engage in the practice of medicine be required to stand an examination before a competent board before being permitted to engage in practice. It matters not what school of medicine he comes from, he should be required to be fully versed in every branch of medicine taught in the school of medicine he claims to be educated in. Now, no educated physician of any school can object to this, unless he wishes to shield ignorance or quackery in some shape or form.

Now is the time to work. The election comes off next November. Let every man work on the candidates for the Legislature during the canvass, and do his whole duty, and in the end we will succeed.

Yours truly,

M. M. MYERS, M. D.

